# Association between erythrocyte Na^+^K^+^-ATPase activity and some blood lipids in type 1 diabetic patients from Lagos, Nigeria

**DOI:** 10.1186/1472-6823-7-7

**Published:** 2007-10-01

**Authors:** Bamidele A Iwalokun, Senapon O Iwalokun

**Affiliations:** 1 Dept of Biochemistry, Lagos State University, PMB. 1087, Apapa-Lagos, Nigeria; 2 Dept. of Endocrinology, Faculty of Clinical Science, Lagos State University, College of Medicine, Ikeja – Lagos, PMB. 21266, Ikeja – Lagos, Nigeria

## Abstract

**Background:**

Altered levels of erythrocyte Na^+^K^+^-ATPase, atherogenic and anti-atherogenic lipid metabolites have been implicated in diabetic complications but their pattern of interactions remains poorly understood.

This study evaluated this relationship in Nigerian patients with Type 1 diabetes mellitus.

**Methods:**

A total of 34 consented Type 1 diabetic patients and age -matched 27 non-diabetic controls were enrolled. Fasting plasma levels of total cholesterol, triglycerides and HDL-cholesterol were determined spectrophotometrically and LDL-cholesterol estimated using Friedewald formula. Total protein content and Na+K+-ATPase activity were also determined spectrophotometrically from ghost erythrocyte membrane prepared by osmotic lysis.

**Results:**

Results indicate significant (P < 0.05) reduction in Na^+^K^+^-ATPase activity in the Type 1 diabetic patients (0.38 ± 0.08 vs. 0.59 ± 0.07 uM Pi/mgprotein/h) compared to the control but with greater reduction in the diabetic subgroup with poor glycemic control (n = 20) and in whom cases of hypercholesterolemia (8.8%), hypertriglyceridemia (2.9%) and elevated LDL-cholesterol (5.9% each) were found. Correlation analyses further revealed significant (P < 0.05) inverse correlations [r = -(0.708-0.797] between all the atherogenic lipid metabolites measured and Na^+^K^+^-ATPase in this subgroup contrary to group with good glycemic control or non-diabetic subjects in which significant (P < 0.05) Na^+^K^+^-ATPase and HDL-C association were found (r = 0.427 - 0.489). The Na^+^K^+^-ATPase from the diabetic patients also exhibited increased sensitivity to digoxin and alterations in kinetic constants Vmax and Km determined by glycemic status of the patients.

**Conclusion:**

It can be concluded that poor glycemic control evokes greater reduction in erythrocyte Na^+^K^+^-ATPase activity and promote enzyme-blood atherogenic lipid relationships in Type 1 diabetic Nigerian patients.

## Background

Diabetes mellitus, a metabolic disease characterized by altered carbohydrate, lipid and protein metabolism and requiring strict glycemic control for delaying or preventing cardiovascular, nephropathic and neurological complications in humans remains a public health problem in developing and developed countries of the world [1]. Several studies in animals and humans have shown that pathogenic course of both Type 1 and Type 2 diabetes mellitus involves alterations in the structures, organization and protein functions of membranes of cells and tissues (e.g. retina, glomerulli, erythrocyte, nerve), culminating in diabetic complications such as retinopathy, nephropathy and peripheral neuropathy [2-4].

Na^+^K^+^-ATPase is a soluble conserved trimeric pump (α, 133 kDa, β, 35 kDa, γ, 10 kDa) involved in transmembrane cation regulation via ATP – dependent efflux and influx of sodium and potassium ions in various cells [5]. The pump is isoenzymic in nature and has its α1 catalytic subunit encoded by the ATP1A1 gene present in both erythrocyte and nerve cells [5,6]. Na^+^K^+^-ATPase has also been reported as one of the membrane proteins affected structurally and functionally in diabetes mellitus [5].

Numerous experimental studies conducted on streptozotocin-induced diabetic rats show that for instance, the delayed motor nerve conduction velocity, decreased nerve blood flow, degeneration of peripheral nerves, persistent hyperglycemia and other metabolic aberrations involve a common denominator of down-regulated Na^+^K^+^-ATPase [4,7,8]. Reduced activity of Na^+^K^+^-ATPase has also been implicated in streptozotocin-induced diabetic rats with nephropathy [9]. De Leo *et al *[10] and Kowluru [11] also reported impairment in the level of this enzyme in diabetic rats and mice with retinopathy. However, in most of these studies, the observed Na^+^K^+^-ATPase reduction was found with other metabolic derangements which include myoinositol depletion, aldolase reduction, sorbitol accumulation and protein kinase C activity [7,12]. Subsequent associations of Na^+^K^+^-ATPase with these parameters have been unfolded [8,12]. Also in humans, several studies have consistently reported a declined Na^+^K^+^-ATPase activity from the membranes of nerve cells and erythrocytes preparations of Type 1 and Type 2 diabetic patients coupled with alterations in membrane protein composition [13-15]. Among diabetic Nigerian patients, defectiveness in Ca^2+ ^homeostasis and qualitative and quantitative changes in erythrocyte membrane proteins have also been reported [16,17]. The work of Okegbile *et al *[18] also revealed a reduction in erythrocyte Na^+^K^+^-ATPase activity in Nigerian patients with Type 2 diabetes mellitus similar to the finding of DeLuise *et al *[19] in obese patients. The latter is characterized by dyslipidemia caused by an alteration in the regulations of cholesterol, triglycerides, high density and low-density lipoproteins [20]. Abnormal levels of these lipids in the blood have subsequently been observed in diabetics with or susceptible to cardiovascular complications and microangiopathy [2].

However, research is still needed to enhance our present understanding about factors responsible for the decreased Na^+^K^+^-ATPase observed in diabetes mellitus especially in Type 1 diabetes mellitus, which occurs early in life and requires life-long insulin therapy [1]. Furthermore, this group of diabetics is also susceptible to cardiovascular complications of diabetes mellitus [1-4] and in our recent study we observed microalbuminuria of comparable prevalence rates in Type 1 and Type 2 diabetic cohorts and in whom elevated blood pressure was also found [21].

Understanding the pattern and magnitude of association between Na^+^K^+^-ATPase activity and cardiovascular risk factors of lipid metabolism would provide a leap forward in our understanding of how diabetes mellitus progresses to cardiovascular complications in patients. Clues to this possibility have emanated from few studies. They include the observation by Rabini *et al *[22] that elevated plasma lysophosphate choline (LPC) due to decreased lecithin-cholesterol acteyltransferase (LCAT) associate strongly with reduced Na^+^K^+^-ATPase activity in diabetic patients. Kiziltunc *et al *[23] also reported cholesterol as an inhibitor of erythrocyte membrane Na^+^K^+^-ATPase in vitro. Inverse correlation between erythrocyte membrane Na^+^K^+^-ATPase activity and polyunsaturated fatty acid levels has also been reported by Djemli-Shipkolye *et al*, [8]. Taken together, there is currently a paucity data on the relationship of Na^+^K^+^-ATPase with cardiovascular risk factors such as total cholesterol, triglycerides and low density lipoprotein cholesterol as well as high density lipoprotein cholesterol. These lipid metabolites are more synonymous with cardiovascular complications than LPC in diabetic patients from Nigeria and other parts of the world 21, 24, 25]. Therefore, knowledge of the metabolic effects of these lipid metabolites on Na^+^K^+^-ATPase activity is essential for a better understanding of the pathogenesis of diabetes mellitus and evolution of strategies that would improve the management and prognosis of the disease.

In the present study, erythrocyte ghost membrane preparations from Nigerian patients with Type 1 diabetes mellitus were evaluated for protein content and Na^+^K^+^-ATPase activity in association with duration of diabetes and their plasma levels of total cholesterol, triglycerides, LDL-cholesterol and HDL-C. The kinetics of the enzyme based on reaction with ATP *in vitro *was also investigated.

## Methods

### Chemicals

All the reagents used in this study were of analytical grade and highest purity. Fatty acid free bovine serum albumin (BSA), sodium dodecyl sulfate (SDS), Tris base and disodium salts of adenosine triphosphate (vanadate free), ethylene diamine tetraacetic acid (EDTA), and Ethyleneglycol – bis-2-aminoethyl ether) N, N, N^1^, N^1 ^– tetracetic acid (EGTA) were purchased from Sigma Chemical Co., St. Louis, MO, U.S.A. Hydrochloric acid (HCl), trichloroacetic acid (TCA), sulphuric acid (H_2_SO_4_), magnesium chloride (MgCl_2_), Deoxycholate, potassium chloride (KCl) and sodium chloride (NaCl) were procured from British Drug House (BDH), UK. All buffers were prepared to their respective pH values measured with the aid of a pH meter (Mettler, Inc. Germany).

### Study design

This study enrolled 34 (male/female, 23/11) Type 1 diabetic out-patients aged 18 – 37 years attending diabetic clinics at General Hospital, Lagos, Nigeria. The patients, diagnosed clinically based on proneness to polydipsia, polyuria, thirst, weight loss and biochemically based on sustained hyperglycemia were placed absolutely on a continuous insulin therapy by attending endocrinologists. A total of twenty-seven apparently healthy non-diabetic volunteers (male/female, 17/10) were also recruited as control. Members in the Type 1 diabetes mellitus group were further subdivided into two subgroups: 'poor glycemic control group' (fasting glucose > 5.6 mmole/L) (group A1) and 'good glycemic control group' (fasting glucose ≤ 5.6 mmole/L) (group A2). The classification was based on WHO treatment guidelines [1]. Fasting blood glucose of members in the control group was also determined twice at three days interval to ascertain their normoglycemic status. Meanwhile, to participate in the study, each member of the study groups had haematocrit and hemoglobin levels greater than or equal to 33% and 11 g/dl respectively, no clinical evidence of pancreatitis and gave written informed consent. Approval to conduct the study was granted by the ethics review committee of the Hospital Management Board. Demographic data including age and duration of diabetics were collected using a questionnaire.

### Sample collection and clinical chemistry

Fasting venous blood samples (5 – 7 mL each) were collected between 8.00 and 9.00 h on each clinic day from the patients and control into labeled heparinized test tubes. The blood samples were centrifuged at 1,500 g at 25°C for 10 min and the resulting plasma transferred into plain test tubes for glucose and lipid analysis.

Fasting plasma glucose was determined using a glucometer (ACCU-CHEK Advantage^®^. Roche Diagnostics Corporation, IND, USA). Plasma cholesterol and triglycerides were determined by the enzymatic method of Siedel et *al *[26] and McGowan *et al *[27] respectively. HCL-cholesterol in plasma was determined following the precipitation of chylomicrons, very low-density lipoproteins (VLDL), intermediate density lipoproteins (IDL) cholesterol and low-density lipoprotein (LDL) cholesterol by MgCl_2 _– phosphotungstic acid mixture as described by Lopez-Virrela *et al *[28]. LDL-cholesterol was estimated according to Friedewald *et al *[29]. Hypercholesterolemia (HCL) was defined as plasma total cholesterol of 200 mg/dl according to the European Artherosclerosis Society guidelines [30]. Hypertriglyceridaemia was put at plasma TAG > 160 mg/dl according to Isselbacher *et al*, [31]. Elevated LDL-C and reduced HDL-C were defined by values > 150 mg/dl and < 35 mg/dl respectively [32].

### Erythrocyte ghost membrane preparation

A simplified procedure of DeLuise and Flier [33] was used for erythrocyte ghost membrane preparation. Briefly, 10 volumes of ice cold 5 mM Tris/0.1 mM Na_2_EDTA, pH 7.6 were added to each of the tubes containing buffy coat free – packed erythrocytes of diabetics and non-diabetic samples to achieve osmotic lysis. The resulting membranes were centrifuged at 20,000 g for 20 min at 4°C. They were then washed three times in 0.017 M NaCl/5 mM Tris-HCl, pH 7.6 and three times with 10 mM Tris-HCl (pH 7.5). The haemoglobin-free membrane suspension was finally stored at -20°C in the 10 mM washing Tris-HCl buffer (pH 7.5) but used within three days of preparation for Na^+^K^+^-ATPase assays and membrane protein determination.

### Na^+^K^+^-ATPase assays

The erythrocyte total ATPase activity was determined by incubating 50 μL of ghost membrane suspension (~200 μg of membrane protein) of type 1 diabetic or healthy subject with 5 mM Tris-ATP, 25 mM KCl, 75 mM NaCl, 5 mM MgCl_2_, 0.1 mM EGTA, 25 mM Tris-HCl, pH 7.5 in a total volume of 500 μL for 90 min at 37°C in a shaking water bath (150 rpm). The reaction was stopped by adding TCA to a final concentration of 5% (wt/vol). After centrifugation for 20 min at 1,500 g, an aliquot of the supernatant was used to measure total inorganic phosphate liberated by the reaction of Fiske and Subbarow [34]. This assay was repeated in the presence of 200 μM methyldigoxin, an inhibitor of Na^+^K^+^-ATPase activity. Total ATPase activity was expressed as micromole of inorganic phosphate liberated per milligram membrane protein per hour. The activity of Na^+^K^+^-ATPase was subsequently determined by subtracting total ATPase activity in the presence of digoxin from enzyme activity in the absence of the inhibitor drug.

For the enzymes kinetics experiment, Na^+^K^+^-ATPase activity was measured in assays containing 0.02 – 0.1 mM Tris-ATP concentration range. A negative control assay devoid of ghost membrane suspension was run in parallel to the test assays and used as blank during absorbance measurement at 660 nm. Enzyme activity was then determined as described previously

The kinetic constants: Vmax and Km were determined by extrapolation from Lineweaver-Burke plot of reciprocally transformed activity (V) and substrate (ATP) data.

### Ghost erythrocyte membrane protein determination

This was determined according to the method of Lowry *et al *[35] after solubilizing aliquots of ghost membrane suspension with 0.2% SDS. Bovine serum albumin (BSA) (50 – 300 μg) was used as standard. Absorbance was measured in a Beckmann D700 spectrophotometer (Beckmann, USA) at 720 nm.

### Statistical analysis

Data obtained was entered into SPSS statistical software version 11 for descriptive and deductive statistics and multivariate regression analyses. All data were expressed as mean ± SD and analyzed by Student's t-test. The metabolic and demographic parameters obtained were subjected to correlation analysis to test their association with erythrocyte membrane (Na^+^K^+^-ATPase activity. P value less than 0.05 was regarded as significant.

## Results

Demographic and haematological characteristics of the diabetic and non diabetic subjects are presented in Table 1. The duration of diabetes ranged from 2 to 8 years with a mean duration of 4.6 years in the type 1 diabetic patients investigated. No significant (P > 0.05) differences were noted between the diabetic patients and non-diabetic control with respect to their age (28.2 ± 3.9 vs. 28.4 ± 4.3 years), haematocrit (39.1 ± 1.4 vs. 39.7 ± 1.2%) and haemoglobin levels (13.1 ± 0.3 vs. 13.2 ± 0.2 g/dL).

**Table 1:** Demographic and haematological characteristics of the study group

Parameter	Diabetics	Control	Statistics
Total number of subjects, n(%)	34 (53.7)	27 (46.3)	> 0.05╠
Number of males, n (%)	23 (67.6)	17 (62.9)	> 0.05╠
Number of females, n (%)	11 (32.4)	10 (37.1)	> 0.05╠
Age, years	28.2 ± 3.9	28.4 ± 4.3	> 0.05╤
^†^Duration of diabetes, years	(2–8) 4.6 ± 1.5	-	ND
Haemoglobin, g/dL	13.2 ± 0.2	13.1 ± 0.3	> 0.05╤
Haematocrit, %	39.1 ± 1.4	39.7 ± 1.2	> 0.05╤

^†^Figures in parenthesis represent range of diabetes duration in the patients; ╠ = Chi-square test (χ^2^); ╤ = Student t- test (Two -tail). P > 0.05 was regarded as non-significant

Compared to the control, significant (P < 0.05) differences were observed in plasma levels of fasting blood glucose (5.5 ± 0.5 vs. 5.0 ± 0.2 mole/L), total cholesterol (182.7 ± 12.2 vs. 173.8 ± 7.4 mg/dL), LDL-C (117.2 ± 14.1 vs. 107.6 ± 6.8 mg/dL), HDL-C (38.1 ± 4.7 vs. 42.2 ± 5.3 mg/dL) and triglycerides (137.1 ± 11.3 vs. 120.4 ± 7.5 mg/dL) in the type 1 diabetic patients investigated. Further stratification of the diabetic patients revealed significant disparity in fasting blood glucose (6.1 ± 0.3 vs. 5.2 ± 0.2 mmole/L), total cholesterol (190.3 ± 15.9 vs. 178.4 ± 6.9 mg/dL), LDL-C (128.1 ± 15.1 vs. 111.0 ± 9.3 mg/dL), HDL-C (33.1 ± 2.6 vs. 40.7 ± 3.5 mg/dL) and triglycerides (145.3 ± 13.3 vs. 133.4 ± 9.7 mg/dL) between patients with poor (n = 20) and good (n = 14) glycemia control. Cases of hypercholesterolemia (3/34, 8.8%), elevated LDL-C (2/34, 5.9%) and hypertriglyceridemia (1/34, 2.9%) were observed in type 1 diabetic patients who had their plasma glucose poorly controlled. Reduced plasma HDL-C levels were observed in 11.1%, 14.7% and 2.9% of the control, poorly and good glycemia control type 1 diabetic subjects studied (Table 2).

**Table 2:** Clinical chemistry profiles of the study group

Parameter	Control	Type 1 diabetic patients		
		A1 (n = 20)	A2 (n = 14)	Total (n = 34)
FBS, mmole/L	5.0 ± 0.2	6.1 ± 0.3^ab^	5.2 ± 0.2	5.5 ± 0.5^a^
TC, mg/dL	173.8 ± 7.4	190.3 ± 15.9^ab^	178.4 ± 6.9^a^	182.7 ± 12.2^a^
LDL-C, mg/dL	107.6 ± 6.8	128.1 ± 15.1^ab^	111.0 ± 9.3	184.2 ± 12.6^a^
HDL-C, mg/dL	42.2 ± 5.3	33.1 ± 2.6^ab^	40.7 ± 3.5	38.1 ± 4.7^a^
TAG, mg/dL	120.4 ± 7.5	145.3 ± 13.3^ab^	133.4 ± 9.7^a^	137.1 ± 11.3^a^
Hypercholesterolemia, n(%)	0(0)	3 (8.8)	0(0)	3 (8.8)
Elevated LDL-C, n(%)	0(0)	2 (5.9)	0(0)	2 (5.9)
Hypertriglyceridemia, n(%)	0(0)	1 (2.9)	0(0)	1 (2.9)
Reduced HDL-C, n(%)	3 (11.1)	5 (14.7)	1(2.9)	6 (17.6)

Data are expressed as mean ± SD and analyzed using Student's t-test (2-tail).

^a^P < 0.05 (control vs. IDDM patients), ^b^P < 0.05 (A1 vs. A2). P <0.05 was considered significant.

Ghost erythrocyte membrane analyses revealed significant (P < 0.05) difference in protein content (5.4 ± 0.2 vs. 4.6 ± 0.4 μg/mL), total ATPase (0.87 ± 0.05 vs. 0.50 ± 0.09 microlePi/mg/h) and Na^+^K^+^-ATPase (0.59 ± 0.07 vs. 0.38 ± 0.08 microlePi/mg/h) activity between the diabetic patients and healthy (non-diabetic) controls. The membrane pump (i.e. Na^+^K^+^-ATPase also exhibited increased sensitivity to digoxin compared to the control (75.1 ± 0.07 vs. 65.9 ± 0.05%; P < 0.05). Further analyses revealed significant (P < 0.05) increases in total ATPase (0.55 ± 0.07 vs. 0.42 ± 0.04 microlePi/mg/h) and Na^+^K^+^-ATPase (0.42 ± 0.07 vs. 0.31 ± 0.04 microlePi/mg/h) activity by 18.7% in Type 1 diabetic patients with good glycemic control compared to their poor glycemic control counterparts. However, the differences observed in the membrane protein content (4.6 ± 0.04 vs. 4.6 ± 0.03 μg/mL) and digoxin sensitivity (74.3 ± 0.06 vs. 75.6 ± 0.07%) between the two diabetic subgroups (A1 and A2) were not significant (P > 0.05) (Table 3).

**Table 3:** Ghost erythrocyte membrane protein content and Na^+^K^+^-ATPase activity

Parameter	Control	Type 1 diabetic patients		
		A1 (n = 20)	A2 (n = 14)	A2 (n = 14)
Protein, μg/mL	5.4 ± 0.2	4.6 ± 0.4^a^	4.6 ± 0.3^a^	4.6 ± 0.4^a^
Total ATPase activity (micromole Pi/mg/h)	0.87 ± 0.05	0.42 ± 0.04^ab^	0.55 ± 0.07^a^	0.50 ± 0.09^a^
Digoxin sensitive Na^+^K^+^-ATPase activity (micromole Pi/mg/h)	0.59 ± 0.07	0.31 ± 0.04^ab ^(52.5)	0.42 ± 0.07^a ^(71.2)^18.7^	0.38 ± 0.08^a ^(64.4)
^‡^Digoxin sensitivity, %	65.9 ± 0.05	75.6 ± 0.07^a^	74.3 ± 0.06^a^	75.1 ± 0.07^a^

Data are expressed as mean ± SD and analyzed using Student's t-test (2-tail). Figures in parentheses represent Na^+^K^+^-ATPase activity in diabetic patients as a percentage of control. Figures in superscripts indicate percentage increase in Na^+^K^+^-ATPase activity in type 1 diabetics with glycemic control over those with poor glycemic control.

^‡^Digoxin sensitivity is defined as percentage decrease in pump activity relative to undigoxinated enzyme^,*a*^P < 0.05 (Control vs. Type 1 diabetic patients), ^b^P < 0.05 (A1 vs. A2). P < 0.05 was considered significant.

**Figure 1:**
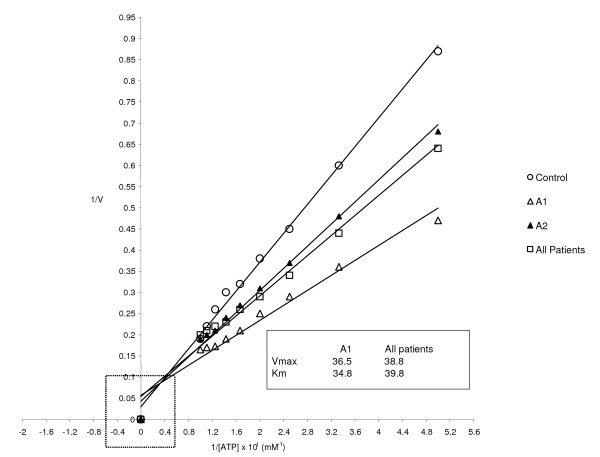
**Lineweaver-Burk plot of erythrocyte membrane Na+K+-ATPase in control and type 1 diabetic patients**. Na+K+-ATPase activity of the ghost erythrocyte membrane at 37°C is plotted as a function of inorganic phosphate released per mg protein per hour. The intercept on the ordinate is equal to 1/Vmax, and the intercept on the abscissa is equal to -1/Km. the method of least squares of means was used to fit data to the Lineweaver-Burk plot. The inset indicates percentage reduction in Vmax and Km values of poor glycemic control (A1) and all diabetic patients compared with type 1 diabetic patients with good glycemic control (A2)

The kinetic profile of erythrocyte Na^+^K^+^-ATPase in the ghost membrane preparations based on reciprocal transformation of data in the Lineweaver-Burk plot is presented in Figure 1. The Na^+^K^+^-ATPase activity with ATP as a substrate exhibited V_max _of 39.2, 26.3, 16.7 and 16.1 micromole Pi/mg/h in control, good glycemic control, poor glycemic and all the diabetic patients respectively. Their respective K_m _values were 0.454, 0.256, 0.167 and 0.154 mM.

Correlation analyses revealed significant (P < 0.05) inverse relationship between FBS (r = -0.784), TC (r = -0.703) and Na^+^K^+^-ATPase activity in all the diabetic patients. In type 1 diabetic patients with poor glycemic control, an additional inverse significant relationship between TAG (r = -0.797), LDL-C (r = -0.707) and membrane enzyme activity was found. These associations were absent in type 1 diabetic patients with good glycemic control.

An exception being significant relationship between HDL–C and enzyme activity (r = 0.427; P = 0.048), similar to observations in non-diabetic control (r = 0.489; P = 0.033) (Table 4).

**Table 4:** Correlation analysis between total Na^+^K^+ ^ATPase activity and other parameters measured in the study group

Na^+^K^+^ATPase.(micromolePi/mg/h)								
	Control		Insulin depedent diabetic patients					
			A1 (n = 20)		A2 (n = 14)		All patients (n = 34)	
FBS	r	P	r	P	r	P	r	P
	-0.18	0.097	-0.963	0.00012*	-0.304	0.07	-0.784	0.044*
Duration of diabetes	-	-	0.173	0.407	0.138	0.425	0.131	0.402
TC	-0.03	0.525	-0.722	0.0043*	-0.582	0.072	-0.703	0.0047*
LDL-C	-0.02	0.454	-0.708	0.0049*	-0.613	0.053	-0.628	0.051
HDL:-C	0.489	0.033*	0.199	0.636	0.427	0.038*	0.275	0.077
TAG	0.216	0.187	-0.797	0.0018*	-0.368	0.092	-0.582	0.063
Protein	0.100	0.279	0.128	0.537	0.147	0.308	0.291	0.274

*Statistically significant at 0.05 level.

## Discussion

Studies conducted to date in humans and animals have consistently shown that diabetes mellitus induces a reduction in erythrocyte membrane Na^+^K^+^-ATPase activity, which results in heamodynamic dysfunction due to altered microvascular blood flow, rheological abnormalities precipitated by decrease erythrocyte defomability and raised fluidity and complications such as nephropathy, neuropathy, cardiovascular disorders and microangiopathy [36,37,1]. The present study has also demonstrated decreased erythrocyte membrane Na^+^K^+^-ATPase activity in Nigerian patients with type 1 diabetes mellitus. Similar findings have been reported in previous studies from other parts of the country involving both type 1 and type 2 diabetic patients [16-18]. However, in these studies the role of glycemic control and metabolic influence of cardiovascular risk factors such as blood pressure on the membrane enzyme activity were not investigated. Meanwhile, studies conducted in other countries have observed decreased enzyme activity in diabetic patients in association with microalbuminuria, hyperkalaemia and elevated serum sialic acid [38,39,14]. In Nigeria, some of these complications have also been found but independently among diabetic patients [21,40]. In the present study, we observed the decreased erythrocyte membrane Na^+^K^+^-ATPase activity to have a significant negative correlation with fasting blood glucose (FBS) and plasma total cholesterol irrespective of the level of glycemic control in all our diabetic patients. Our observation with respect to FBS is supportive of previous findings in which glucose in diabetic state exhibited direct toxicity on erythrocyte membrane protein including Na^+^K^+^-ATPase through non-enzymatic glycosylation [41,42]. Although, the level of glycosylated hemoglobin (HbA1c) was not determined in our diabetic cohort, the greater FBS-Na^+^K^+^-ATPase association observed in our patients with poor glycemic control is suggestive of advanced glycation of the enzyme since this non-enzymatic process has been associated with greater reduction in Na^+^K^+^-ATPase activity and other cellular signaling proteins such as protein kinase C owing to their proneness to glycability [43]. The greater reduction in Na+K+ATPase observed among our type 1 diabetic subgroup with poor glycemic control may also be due to greater susceptibility of this protein to oxidative stress [44]. The fact that glycation can be minimized through glycemic control as demonstrated in previous studies [9], there is huge possibility of reducing the magnitude of Na^+^K^+^-ATPase inevitable reduction and subsequent complications in diabetic patients in order to reduce morbidity and mortality rates, currently on the increase in Nigeria [45]. The poor glycemic control observed in 20 of the 34 patients investigated may not be unconnected with poor compliance to insulin therapy, the use of which is determined in part by socioeconomic factors which include poverty, access to treatment and quality of care in most developing countries [21]. Our observation of the plasma total cholesterol as the only cardiovascular risk factor to correlate negatively with Na^+^K^+^-ATPase activity in our diabetic cohort irrespective of glycemic control is indicative of an exceeding clinical importance of this lipid metabolite in type 1 diabetic patients. Earlier studies in this regard are mostly *in vitro *in which decreased erythrocyte membrane cholesterol content and cholesterol:phospholipid ratio were found in erythrocyte ghost preparations from diabetic patients [8,46]. Since cholesterol modulates erythrocyte membrane fluidity, its loss into systemic circulation may therefore contribute to the increased fluidity observed in diabetic patients [37]. However, there is a strong indication from this study and previous ones that poor glycemic control may exacerbate the reported cholesterol loss, which when added to the contributions from diet and endogenous synthesis may increase the risk of hypercholesterolemia in diabetes and this we have found only in our IDDM patients with poor glycemic control. The same explanation can be adduced for the incidence of hypertriglyceridemia and elevated LDL-cholesterol in this group of type 1 diabetic patients since they also exhibited significant Na^+^K^+^-ATPase – TAG/LDL-C association not found in their good glycemic control counterparts. The observed significant positive relationship between enzyme activity and plasma HDL-cholesterol in normal and type 1 diabetic patients with good glycemic control validates not only the anti-atherogenic function of this lipid metabolites but also implicates its presence as a protective factor against cardiovascular complications in this subgroup. It raises the possibility of using glycemic control as a prerequisite for achieving lipid control in type 1 diabetic patients as recently reported by Erciyas *et al *[47]. Our observed lack of association between duration of diabetes, membrane protein and Na^+^K^+^-ATPase activity is in agreement with previous findings [3]. However, Adewoye *et al *[17] had previously reported changes in erythrocyte membrane protein composition in diabetic Nigerians to involve loss of spectrin and preponderance of ankyrin. The erythrocyte membrane protein content of 4.3 – 5.1 μgmL in diabetics and 5.5 μg/mL in healthy humans reported by these workers are comparable to our findings and thus validate their results. In this study, our non-diabetic controls were matched for age with the diabetic groups to minimize the impact of age on Na^+^K^+^-ATPase activity and validate of the results obtained. Also, the effect of hematological abnormalities on Na^+^K^+^-ATPase activity [48] was annulled by inclusion of subjects with hematocrit level ≥ 33% and hemoglobin level of 11 g/dL and above.

We also observed reduced Vmax for Na^+^K^+^-ATPase from diabetic erythrocyte ghost membrane preparations compared to the control using ATP as the substrate. Also, greater reduction in the kinetic constant was observed in poor glycemic control patients. Our data are in concordance with the finding of Okegbile *et al *[18] in type 2 diabetic patients. Reduced Vmax connotes a reduction in the enzyme ability to hydrolyze ATP *in vitro*, which undoubtedly has physiological implications in the regulation of transmembrane cation transport in the erythrocytes. While diabetes may be a factor for this event, numerous secondary factors have been reported. They include decreased number of pump units on the erythrocyte membrane, altered lipid – protein interaction, depleted membrane anionic charge and enzyme glycation and peroxidation [9,10,20,42]. We also observed lower Km values for the membrane enzyme in our diabetic patients compared to the control and this suggest increased affinity for ATP *in vitro*. This observation is contrary to what Okegbile *et al *[18] observed in type 2 diabetic patients. However, Okegbile *et al *[18] conducted their kinetic assay in the presence of digoxin and this may explain the difference in our observations. In this study, assays conducted in the presence of digoxin were carried out to compare the sensitivity of Na^+^K^+^-ATPase from both diabetic and non-diabetic subjects to this digitalis. We found the diabetic enzyme to exhibit an increased sensitivity ranging from 74.3 – 75.6% compared to 69.5% in the non-diabetic control. This indicates a greater inhibition of diabetic Na^+^K^+^-ATPase enzyme by digoxin similar to observations with Ouabain [49].

Based on the results of this study, we conclude that the reduced erythrocyte Na^+^K^+^-ATPase activity which occurs in type 1 diabetes mellitus is exacerbated in Nigerian patients with poor glycemic control in association with atherogenic blood lipids.

## Competing interests

The author(s) declare that they have no competing interests.

## Authors' contributions

BAI – Conceived the idea of the study, wrote the proposal for ethical approval (with clinical inputs from SOI), provided the literature, did the laboratory analyses and wrote the manuscript.

SOI is in charge of patient recruitment, drafting of the consent form and administration of the questionnaire, manuscript editing and input.

Both authors read and approved the final manuscript.

## Pre-publication history

The pre-publication history for this paper can be accessed here:


